# Modifications of *Pseudomonas aeruginosa* cell envelope in the cystic fibrosis airway alters interactions with immune cells

**DOI:** 10.1038/s41598-017-05253-9

**Published:** 2017-07-06

**Authors:** Preston J. Hill, Julia M. Scordo, Jesús Arcos, Stephen E. Kirkby, Mark D. Wewers, Daniel J. Wozniak, Jordi B. Torrelles

**Affiliations:** 1 Department of Microbial Infection and Immunity, College of Medicine, The Ohio State University, Columbus, OH 43210 USA; 2 Nationwide Children’s Hospital, Section of Pulmonary Medicine, Columbus, OH 43205 USA; 3 Department of Internal Medicine, Pulmonary, Critical Care and Sleep Medicine Division, College of Medicine, The Ohio State University, Columbus, OH 43210 USA; 4 Center for Microbial Interface Biology, The Ohio State University, Columbus, OH 43210 USA; 5 Department of Microbiology, The Ohio State University, Columbus, OH 43210 USA

## Abstract

*Pseudomonas aeruginosa* is a ubiquitous environmental organism and an opportunistic pathogen that causes chronic lung infections in the airways of cystic fibrosis (CF) patients as well as other immune-compromised individuals. During infection, *P*. *aeruginosa* enters the terminal bronchioles and alveoli and comes into contact with alveolar lining fluid (ALF), which contains homeostatic and antimicrobial hydrolytic activities, termed hydrolases. These hydrolases comprise an array of lipases, glycosidases, and proteases and thus, they have the potential to modify lipids, carbohydrates and proteins on the surface of invading microbes. Here we show that hydrolase levels between human ALF from healthy and CF patients differ. CF-ALF influences the *P*. *aeruginosa* cell wall by reducing the content of one of its major polysaccharides, Psl. This CF-ALF induced Psl reduction does not alter initial bacterial attachment to surfaces but reduces biofilm formation. Importantly, exposure of *P*. *aeruginosa* to CF-ALF drives the activation of neutrophils and triggers their oxidative response; thus, defining human CF-ALF as a new innate defense mechanism to control *P*. *aeruginosa* infection, but at the same time potentially adding to the chronic inflammatory state of the lung in CF patients.

## Introduction

*Pseudomonas aeruginosa* is ubiquitous and especially common in humid environments and in the endogenous flora of hospitalized individuals^1, 2^. *P*. *aeruginosa* causes devastating infections in individuals with the genetic disease cystic fibrosis (CF) resulting in significant morbidity and mortality^3, 4^. A critical aspect of this infection involves the formation of microbial communities called biofilms^5^. *P*. *aeruginosa* biofilm growth within the lungs of individuals with CF complicates this disease by adding to the chronic inflammatory state of the lung. In contrast to planktonic cells, bacterial biofilms are characterized by their antimicrobial recalcitrance as well as by conferring resistance against the host immune response.

*P*. *aeruginosa* synthesizes special cell wall properties that allow the formations of biofilms, which can be visualized in individuals with CF^6, 7^. *P*. *aeruginosa* biofilms are characterized by the presence of an extracellular matrix^8–10^ containing polysaccharides, proteins, and extracellular DNA. These polysaccharides are important in biofilm initiation, development and maintenance^11^. *P*. *aeruginosa* produces three major polysaccharides: alginate, Pel, and Psl. *P*. *aeruginosa* variants expressing alginate are mucoid^12^, which is a hallmark of an established, biofilm-mode of infection. Non-mucoid variants of *P*. *aeruginosa* may contain two additional polysaccharides, Psl and Pel^13^. Pel is critical to biofilm architecture by maintaining interactions among cells^14^. Psl is instrumental in biofilm initiation, developing the structure of the biofilm and participating in the evasion of the innate immune response by blocking complement deposition and reducing the neutrophil oxidative response^15^. The two most common *P*. *aeruginosa* non-mucoid laboratory strains studied are PAO1 and PA1^14^. PAO1 expresses Psl on its cell surface, along with minimal amounts of Pel, while strains such as PA14 express Pel but are devoid of Psl^14, 16–18^.

In individuals with CF, *P*. *aeruginosa* establishes chronic infections throughout different regions of the lung, *i*.*e*. upper, middle and lower lung regions^19^. Additionally, *P*. *aeruginosa* isolates differ phenotypically and evolve independently within the CF lung based on their regional location^19^. This suggests that regional environmental pressures, such as the alveolar mucosa surrounding the alveolar space of the lungs^9, 20^ may impact *P*. *aeruginosa* in individuals with CF. The alveolar mucosa is obtained by bronchoalveolar lavage upon deposition of the bronchoscope close to the entry of the bronchi, allowing for washing of the bronchioles and alveoli. Alveoli contain the majority of the washed surface area [75 (116,250 sq. inches) to 100 (155,000 sq. inches square) meters squared]^21^; therefore, the recovered fluid (alveolar mucosa) contains mainly alveolar components^22^. Alveolar mucosa can be separated into two distinct components, the alveolar lining fluid or ALF (hypophase) and the surfactant (lipidic phase)^22^. Surfactant and ALF are produced and secreted by type II alveolar epithelial cells^22, 23^. ALF contains numerous hydrolytic activities (called hydrolases), which play a regulatory role in antimicrobial pathways and maintenance of alveolar homeostasis^24^. These hydrolases are hypothesized to alter key properties of microbial cell surfaces and modify specific host-pathogen interactions^25^. Our studies with *Mycobacterium tuberculosis* (*M*.*tb*) demonstrate that these hydrolases modify the *M*.*tb* cell wall properties significantly reducing the surface exposure of two of the major cell wall factors involved in *M*.*tb* intracellular survival, mannose-capped lipoarabinomannan and trehalose dimycolate^26^. *M*.*tb* exposure to human ALF also altered its interaction with and infection outcome in host phagocytes^26–29^.

The role of ALF hydrolases play in acute and chronic CF airway infection and inflammation is poorly understood. We hypothesize that upon microbial deposition in the alveolar space, ALF hydrolases have the potential to remodel the *P*. *aeruginosa* cell envelope prior to its interaction with host alveolar compartment cells. Our results show that the most bioactive CF-ALF hydrolases are acid phosphatase and α-mannosidase. These hydrolases can alter *P*. *aeruginosa* cell wall properties. We observed that these cell wall alterations are predominant in *P*. *aeruginosa* strains expressing Psl exopolysaccharide on their cell surface. In addition to its role in biofilms^30^, planktonic *P*. *aeruginosa* also produces Psl which promotes surface attachment by acting as ‘molecular glue’^31^. After this event, individual surface-attached bacteria self-organize into microcolonies which is the first step in communal biofilm organization (the mechanism behind this process is described elsewhere)^32^. Thus, we further determined that CF-ALF hydrolases alter the surface expression of Psl in planktonic *P*. *aeruginosa* and diminish biofilm formation capability. Importantly, *P*. *aeruginosa* exposure to CF-ALF also reduces the ability of *P*. *aeruginosa* to be opsonized, as well as alters the neutrophils immune response by increasing their activation status and oxidative response, which could contribute to the successful establishment of the infection by inducing tissue damage. Here we define ALF hydrolases in individuals with CF that may influence the successful establishment of the *P*. *aeruginosa* infection.

## Results

### Enzymatic activities of hydrolases present in human ALF from CF patients

**Figure 1:**
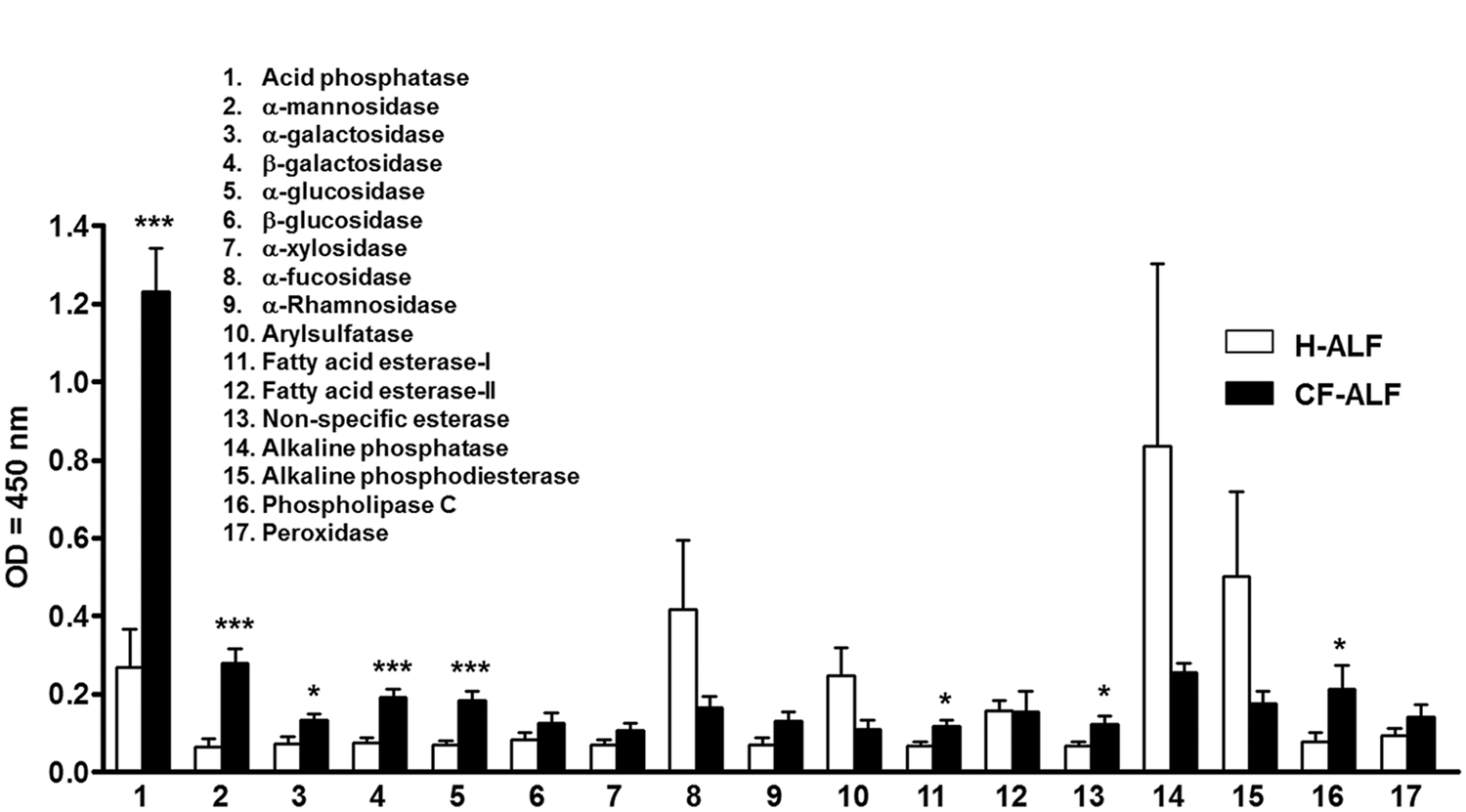
Basal expression of several hydrolase activities in CF-ALF and H-ALF. Hydrolase activities present in CF-ALF (n = 10, in triplicate, M ± SEM) and H-ALF (n = 14, in triplicate, M ± SEM) were monitored using a colorimetric method based on the release of *p*-nitrophenol upon specific substrate cleavage. Results show the presence of several hydrolases with different levels of activity that have the potential to remodel the cell envelope of *M*.*tb* within the alveolar environment. 1. Acid phosphatase; 2. α-mannosidase; 3. α-galactosidase; 4. β-galactosidase; 5. α-glucosidase; 6. β-glucosidase; 7. α-xylosidase; 8. α-fucosidase; 9. Arylsulfatase; 10. Fatty acid esterase-I; 11. Non-specific esterase; 12. Alkaline phosphatase; 13. Alkaline phosphodiesterase; 14. Phospholipase C; 15. Peroxidase; 16. α-Rhamnosidase; and; 17. Fatty acid esterase-II. Student’s *t*-test, H-ALF *vs*. CF-ALF, unpaired, two-tailed, *p < 0.05; **p < 0.005; ***p < 0.0005; for each ‘n’ value, an ALF from different donor was used.

To determine which hydrolases with the potential to alter the *P*. *aeruginosa* cell envelope are present in the human alveolar space, we obtained ALF in 0.9% NaCl from healthy donors (n = 14) and CF individuals (n = 10) at their *in vivo* relevant concentrations of 1.0–1.5 mg/phospholipids as determined by a phosphate assay^23, 33^ as we previously described^26^. A colorimetric assay based on the cleavage of *p*-nitrophenol from a specific substrate was performed to measure hydrolase activities that could alter the *P*. *aeruginosa* cell wall^26^. The activities of 17 hydrolases from ALF are summarized in Fig. 1. Our results (normalized to negative controls) show evidence of hydrolase activities present in human ALF. Specifically, a significant increase of the enzymatic activities of acid phosphatase, α-mannosidase, α- and β-galactosidase, α-glucosidase, phospholipase C, and fatty acid esterase-I and -II was observed in ALF from CF individuals (CF-ALF), when compared to ALF from healthy donors (H-ALF). Compared with H-ALF, there was a decrease, although not statistically significant, of α-fucosidase, arylsulfatase, and both alkaline phosphatase and alkaline phosphodiesterase in the CF-ALF.

### Effect of hydrolase activities derived from CF-ALF on live *P*. *aeruginosa*

We reasoned that upon deposition into the alveolar space, *P*. *aeruginosa* will be exposed to the basal levels of these hydrolytic enzymes, and thus, potentially these could alter the *P*. *aeruginosa* cell wall prior encountering host alveolar compartment cells. To assess if hydrolases in CF-ALF were capable of modifying the *P*. *aeruginosa* cell envelope *in vivo*, bacteria were harvested and exposed to CF-ALF normalized at physiological concentration *in vivo* [1 mg of phospholipid/ml]^26^ from 3 different donors, commercial acid phosphatase and α-mannosidase (at the levels determined in Fig. 1), or 0.9% NaCl as control.

**Figure 2:**
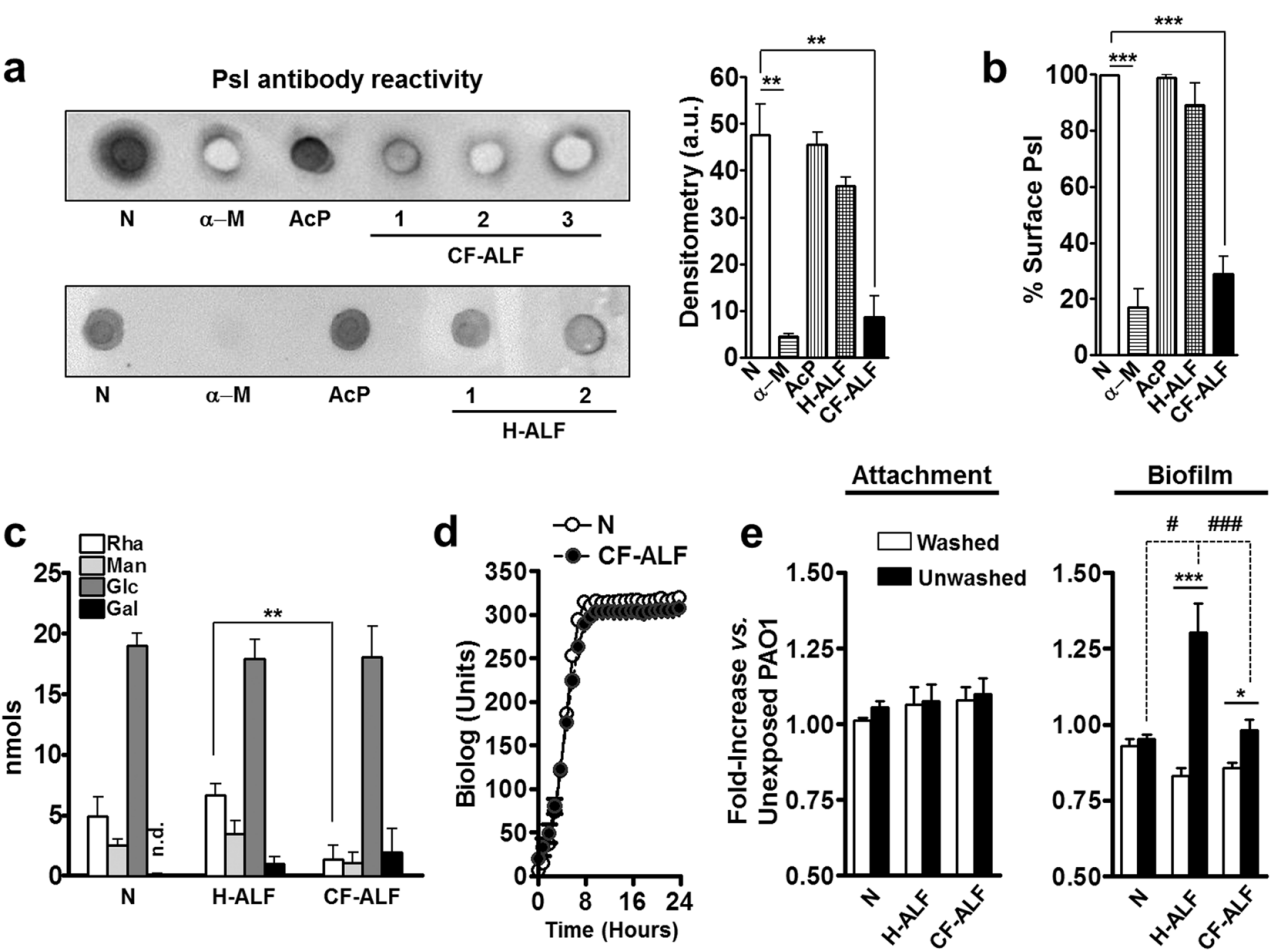
CF-ALF reduces the *P*. *aeruginosa* cell surface Psl. (**a**) Psl is altered by hydrolases present on CF-ALF. Psl was extracted from *P*. *aeruginosa* strains previously exposed to 0.9% NaCl (N, control), acid phosphatase (AcP), α-mannosidase (α–M) at their physiological concentration within the CF lung, or directly exposed to CF-ALF from 3 different individuals (denoted on dot blot as CF-ALF 1, 2, 3) or H-ALF from 2 different individuals (denoted on dot blot as H-ALF 1, 2). Extracted Psl was spotted on membranes and dot blots performed to quantify the total amount of Psl remaining on *P*. *aeruginosa* strains after exposure to the above conditions. Results indicate that CF-ALFs and α–M modify the *P*. *aeruginosa* cell wall by reducing Psl as indicated by the loss of anti-Psl immunoreactivity. Student’s *t*-test, unpaired, two-tailed, **p < 0.005. (**b**) Psl is reduced from the bacterial cell surface of *P*. *aeruginosa* exposed to CF-ALF. Whole cell ELISA using the *P*. *aeruginosa* PAO1 strain exposed to different CF-ALF showing the reduction of Psl on the cell surface. Student’s *t*-test, unpaired, two-tailed, **p < 0.005, n = 3. (**c**) Carbohydrate composition analysis of CF-ALF-exposed *P*. *aeruginosa*. *P*. *aeruginosa* (2 × 10^7^) was exposed to 0.9% NaCl (control) or human H-ALF or CF-ALF. Alditol acetates obtained from exposed-whole bacteria were further analyzed by GC/MS based on bacterial numbers. Shown are cumulative data of n = 3 each performed in duplicate (M ± SEM) and using different H-ALF and CF-ALF in each experiment; rhamnose (Rha), mannose (Man), glucose (Glc) and galactose (Gal). Student’s *t*-test, unpaired, two-tailed, **p < 0.005. (**d**) *P*. *aeruginosa* exposed to CF-ALF retains its ability to grow extracellulary. *P*. *aeruginosa* was exposed to 0.9% NaCl or CF-ALF. Results show that CF-ALF does not impair the ability and capability of *P*. *aeruginosa* to grow extracellularly, n = 2. (**e**) *P*. *aeruginosa* exposed to CF-ALF retains its capacity to attach to plastic surfaces but decreases its capacity to form biofilms. Student’s *t*-test, *P*. *aeruginosa* exposed to 0.9% NaCl (control) *vs*. exposed to H-ALF *vs*. exposed to CF-ALF, unpaired, two-tailed, *p < 0.05; ***p < 0.0005 (between white bars), ^§^ *p* < 0.05 (between black bars), ^##^ *p* < 0.05, ^###^ *p* < 0.005 (between white and black bars), n = 3 for 0.9% NaCl and H-ALF exposure, and n = 7 for CF-ALF exposure. In all the experiments, for each ‘n’ value, a H-ALF and CF-ALF from different donors were used.

Since the exopolysaccharide Psl is one of the major exopolysaccharide on the cell surface of non-mucoid *P*. *aeruginosa* ^13^, we focused our attention on assessing if hydrolases present in CF-ALF impact its levels. Dot blot of the surface extracted Psl in Fig. 2a followed by densitometry analysis indicates that *P*. *aeruginosa* exposure to CF-ALF significantly reduces the amount of bacterial surface associated Psl by ∼82.0%; in contrast, exposure to H-ALF reduced the Psl on the *P*. *aeruginosa* surface by only 22.5%. ELISA further confirmed these results, where we observed a similar trend, a 71.2% % cell surface reduction of Psl after *P*. *aeruginosa* exposure to CF-ALF (Fig. 2b) and only 10.9% reduction when exposed to H-ALF. This reduction was also observed in bacteria exposed to α–mannosidase (90.7% by densitometry and 83% by ELISA) but not when exposed to acid phosphatase (1 and 4% by ELISA and densitometry, respectively), indicating that the significant high levels of α–mannosidase observed in CF-ALF may directly contribute to the loss of Psl immunoreactive motifs observed on the bacterial cell surface.

The Psl structure was identified as repeating units of a neutral, branched pentasaccharide consisting of D-glucose (Glc), D-mannose (Man) and L-rhamnose (Rha) monosaccharides^34^. The biochemical alteration of the *P*. *aeruginosa* cell wall and effects on Psl were thus further corroborated by our sugar analysis on whole bacteria exposed to CF-ALF, demonstrating a reduction in the overall content of Rha and Man in CF-ALF exposed bacteria compared to our control 0.9% NaCl-exposed bacteria, as well as when compared to bacteria exposed to H-ALF (Fig. 2c). Unexpectedly, the overall cell wall content of Glc remained unaffected. Importantly, these differences in total carbohydrate content were only observed in CF-ALF exposed *P*. *aeruginosa*, as bacteria exposed to H-ALF did not have alterations in their overall cell wall sugar content when compared to control (0.9% NaCl-exposed bacteria) (Fig. 2c).

As *P*. *aeruginosa* exposure to CF-ALF results in bacterial cell wall alterations, we further assessed if *P*. *aeruginosa* exposure to CF-ALF impaired bacterial growth. Results in Fig. 2d indicate that CF-ALF exposed *P*. *aeruginosa* grew at similar rate to our control (0.9% NaCl exposed) bacteria. These results indicate that *P*. *aeruginosa* exposure to CF-ALF does not kill *P*. *aeruginosa* and does not significantly impair growth.

### Effects of CF-ALF on *P*. *aeruginosa* adhesion and biofilm formation

Our above results indicate that exposure of *P*. *aeruginosa* to CF-ALF has two consequences: modifications on the *P*. *aeruginosa* cell wall surface and release of cell wall fragments into the milieu. We next determined if exposure to CF-ALF alters the capacity of *P*. *aeruginosa* to attach to surfaces, and thus, establish a biofilm. In this assay we used *P*. *aeruginosa* PAO1, characterized for having abundant Psl on its cell surface, and as a control *P*. *aeruginosa* WFPA800, which lacks Psl. We assessed both, if the induced cell wall modifications alone (washed, white bars) or together with the released material (unwashed, black bars) affected *P*. *aeruginosa* capacity to attach (2 hours assay) or form a biofilm (overnight assay).

Results in Fig. 2e indicate that PAO1 strain similarly attach to plastic surfaces independently of both, being exposed to 0.9% NaCl, H-ALF or CF-ALF and the presence of the released cell wall material (white *vs*. black bars). Interestingly, we found that under the unwashed conditions (black bars); H-ALF exposed *P*. *aeruginosa* was more efficient in forming biofilms. However, under the washed conditions (white bars), H-ALF and CF-ALF exposed *P*. *aeruginosa* had reduced ability to form biofilms compared to 0.9% NaCl exposed bacteria. Under all conditions studied, our WFPA800 control strain lacking Psl presented deficient attachment and biofilm formation (data not shown).

### Impacts of CF-ALF on *P*. *aeruginosa* opsonization and subsequent interactions with neutrophils

**Figure 3:**
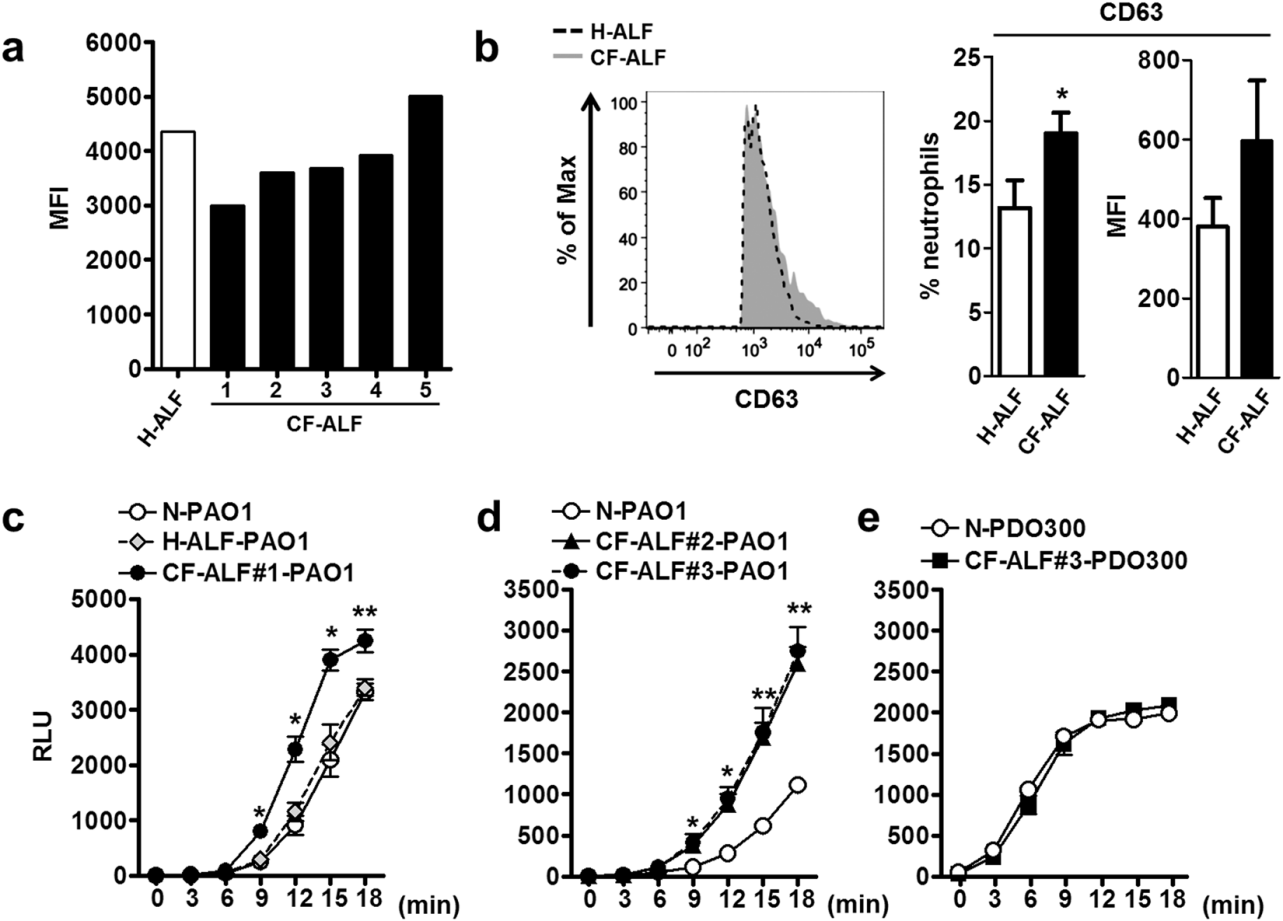
Effects of *P*. *aeruginosa*-exposed ALFs on innate immune functions. (**a**) A representative experiment (of n = 3) of mean fluorescent intensity (MFI) of serum C3 binding to *P*. *aeruginosa*, with each ‘n’ value representing a different human neutrophil donor. Non-mucoid PAO1 exposed to CF-ALF show a decrease in serum C3 deposition in 4 of 5 tested CF-ALF samples, with each CF-ALF representing a different human donor. For all three experiments a total of 3 different healthy ALFs (one per each n value) and 7 different CF ALFs (distributed among the n values) were used. (**b**) Neutrophil activation and degranulation are enhanced when infected with CF-ALF exposed *P*. *aeruginosa*. Following infection with H-ALF or CF-ALF exposed *P*. *aeruginosa*, CD63 surface expression, a marker of neutrophil activation and primary granule release, on neutrophils was detected by flow cytometry. A representative histogram (of n = 4) showing the CD63 surface expression increase on neutrophils following infection with CF-ALF-exposed *P*. *aeruginosa*. Black dashed line represents healthy ALF-exposed *P*. *aeruginosa* and solid grey represents CF ALF-exposed *P*. *aeruginosa* (left). Percentage and MFI of neutrophils expressing CD63 following infection with H-ALF-exposed or CF-ALF-exposed *P*. *aeruginosa*. Data shown are n = 4; M + SEM (right). Each ‘n’ value represents a different human neutrophil donor and a total of 4 healthy and 4 CF ALFs were used in these experiments. Student *t* test, *P*. *aeruginosa* exposed to H-ALF *vs*. exposed to CF-ALF, *p < 0.05. (**c**,**d**) CF ALF exposures enhance ROS burst response of neutrophils exposed to non-mucoid *P*. *aeruginosa*. Neutrophils were infected with CF-ALF pre-exposed *P*. *aeruginosa* PAO1 strain. Results indicate a significant increase of ROS production, representative experiments performed in triplicate. (**e**) This phenomenon was not observed in in the mucoid strain (PDO300), representative experiment in triplicate. In (**c**–**e**), Student t test, *P*. *aeruginosa* exposed to 0.9% NaCl (control) *vs*. exposed to CF-ALF, *p < 0.05; **p < 0.005. N-PAO1: PAO1 exposed to 0.9% NaCl; H-ALF-PAO1: PAO1 exposed to ALF from a healthy individual; CF-ALF#1- or #2- or #3-PAO1: PAO1 exposed to CF-ALF from different individuals; N-PDO300: PDO300 exposed to 0.9% NaCl; CF-ALF-DPO300: PDO300 exposed to CF-ALF.

To determine if *P*. *aeruginosa* exposure to CF-ALF affects the innate immune response, we first determined if exposure to H-ALF or CF-ALF affects opsonization of *P*. *aeruginosa*. This was evaluated by directly measuring serum complement component 3 (C3) deposition on the bacterial surface by flow cytometry. Compared with exposure to H-ALF, *P*. *aeruginosa* exposure to CF-ALF resulted in a reduction in serum C3 bacterial opsonization (observed in 4 out of 5 CF-ALF samples studied) (Fig. 3a). Next we determined if H-ALF or CF-ALF altered the activation status of resting neutrophils. Our results indicate that *P*. *aeruginosa* exposed to CF-ALF significantly increased the activation status of neutrophils (increased expression of CD63 on the surface of resting neutrophils indicative of primary degranulation and neutrophil activation) when compared to *P*. *aeruginosa* exposed to H-ALF (Fig. 3b). However, significant changes of CD66b (marker of secondary degranulation) or CD11b (CR3) were not observed (data not shown). We further assessed the oxidative response of neutrophils infected with non-mucoid (PAO1 strain) and mucoid (PDO300 strain) *P*. *aeruginosa* variants pre-exposed to CF-ALF and H-ALF. Results in Fig. 3c indicate that *P*. *aeruginosa* exposed to CF-ALF induces a significantly stronger production and release of neutrophil’s radical oxygen species (ROS). This phenotype was further corroborated with CF-ALF from multiple donors (Fig. 3d). Interestingly, this phenotype was only observed in the non-mucoid *P*. *aeruginosa* variant (PAO1) known to express Psl on its cell wall surface, as CF-ALF exposed mucoid *P*. *aeruginosa* variant expressing alginate (PDO300) did not further increase the release of ROS by neutrophils (Fig. 3e).

## Discussion

*P*. *aeruginosa* is the causative agent for one of the most prevalent microbial infections in CF patients^3, 4^. Upon deposition in the lung, *P*. *aeruginosa* isolates are capable of establishing infection in different regions of the lung^19^. Differential evolution and functionality of these isolates suggest that the existing multiple microenvironments in the lung may impact *P*. *aeruginosa* infection. In this regard, *P*. *aeruginosa* deposited into the alveolar space is in close contact with ALF and its hydrolytic enzymes. The main function of ALF hydrolases is to assist in the recycling and degradation of the alveolar mucosa^35^. These ALF hydrolases may be altered due to the inflammation caused by CF and/or by microbial infection^36^. Our current results suggest that the lung airways in CF patients may harbor hydrolases that benefit a chronic *P*. *aeruginosa* infection, directing the robust neutrophil-dominant airway inflammation^37^.

Based on the biochemical composition of the *P*. *aeruginosa* cell wall, we assessed the bioactivity of a series of hydrolases present in the lung. Our results indicate a significant increase of acid phosphatase (∼4.6-fold increase) in the lung milieu of CF patients. This high level of acid phosphatase has been also measured in patients with pulmonary sarcoidosis and cryptogenic fibrosing alveolitis (also known as idiopathic pulmonary fibrosis)^38^. The role of acid phosphatase in microbial infections is not clear, although it is likely that this lipid phosphohydrolase has critical roles in lung morphogenesis and in acute lung injury and repair^39^. The presence of acid phosphatase has been previously described in mammalian ALF^40^, where this enzyme contains characteristics of the microsomal enzyme, and it is quite specific for the hydrolysis of phosphatidic acid and 1-acyl-2-lysophosphatidic acid. An increase of acid phosphatase in CF-ALF may correspond to the homeostatic necessity to quickly degrade accumulation of lysophospholipids, which are related to surfactant dysfunction and lung inflammation^41^. The second most bioactive enzymatic activity found in CF-ALF is α-mannosidase. Although the exact function of this hydrolase in the alveolar mucosa is not clear, studies showed that the lung α-mannosidase originates in epithelial cell type II lamellar bodies and is a lysosomal-type hydrolase with optimum activity at acidic pH^42^. In this regard, CF-ALF is acidic^43^. What implication this may have in *P*. *aeruginosa* infection is not clear, however, ALF with acidic pH also has impaired antibacterial killing capacity^43^.

Importantly, our results indicate that CF-ALF also contains significant amounts of phospholipase C and fatty acid esterases. Dipalmitoylphosphatidylcholine (DPPC), the major phospholipid of human ALF surfactant, is degraded by *P*. *aeruginosa* phospholipase C^44^ and extracellular lipases and fatty acid esterases to produce glycerol and free fatty acids. DPPC can be used by *P*. *aeruginosa* as a sole source of carbon, nitrogen, and phosphorous for growth^44, 45^, and *P*. *aeruginosa* growth in the presence of DPPC leads to the emergence of mucoid variants with elevated levels of alginate^46^. Thus, the increase of fatty acid esterases in CF-ALF may facilitate DPPC degradation for use as a *P*. *aeruginosa* nutrient source within the CF lung. Furthermore, DPPC-derived glycerol (by the action of CF-ALF fatty acid esterases) could be a particular good source of carbon for alginate biosynthesis within the CF lung, as it has been shown that *P*. *aeruginosa* growth in medium containing high-concentrations of glycerol promotes the appearance of mucoid variants from non-mucoid bacteria^45^.

The effects of CF-ALF on the *P*. *aeruginosa* cell wall were obvious in the PAO1 strain, which is well known to expose Psl on its cell surface. Our results indicate that modifications of Psl or potential release of Psl into the milieu may occur after *P*. *aeruginosa* is exposed to CF-ALF, and at lesser extend to H-ALF. Based on the Psl structure and the hydrolases detected in CF-ALF, a decrease in mannose and rhamnose was observed, but not in glucose. As Psl is formed by these three monosaccharides, a plausible explanation for this observation is that the majority of the enzymatic activity detected in ALF that could alter Psl have exoglycosidase activity, *i*.*e*. α-mannosidase, α-rhamnosidase, α-glucosidase and β-glucosidase; which imply the release of terminal residues or short oligosaccharides from Psl. Psl is formed by a penta-saccharide repeating unit which consist of a lineal core β-Glc*p*(1-3)-β-Man*p*(1-3)-β-Man*p*-(1-3)-α-Rha*p* with a single terminal α-Man*p* branching from the C2 position of the first 3-β-Man*p* ^34^. Thus, the high content of α-mannosidase present in CF-ALF could remove Psl single branched terminal 2-α-Man*p* residues (highly present in Psl). Additionally, the levels of α-rhamnosidase detected in CF-ALF could also remove the Psl terminal 3-α-Rha*p*, as well as, remove rhamnose from the *P*. *aeruginosa* LPS^47^ and rhamnolipid^48^. However, our sugar analysis also showed no variation in glucose residues, indicating that the β-glucosidase present in H-ALF and CF-ALF has limited or no access to the Psl terminal 1-β-Glc*p*. Endoglycanase activities that could internally cleave Psl and other *P*. *aeruginosa* polysaccharides such as alginate, Pel, rhamnolipid and two types of lipopolysaccharide (LPS), were not detected in ALF (data not shown). To which extent these *P*. *aeruginosa* components are altered by *P*. *aeruginosa* exposed to CF-ALF or H-ALF, and its implications in host cell recognition is currently being studied in the lab.

Unexpectedly, changes on Psl on the ALF exposed *P*. *aeruginosa* did not translate in differences in PAO1 adhesion to cell surfaces but influenced the capacity to form biofilms (observed for both cases, H-ALF and CF-ALF exposure). This result is consistent with the literature, which shows lack of Psl (using a *P*. *aeruginosa* mutant completely devoid of Psl) is directly related to decrease in biofilm formation^49–51^. The fact that biofilm formation is reduced but not completely eliminated could be a consequence that CF-ALF exposure only partially reduces Psl (by ∼82%) on *P*. *aeruginosa*; thus, the remaining Psl present after exposure to CF-ALF (∼18%) might be sufficient for *P*. *aeruginosa* to maintain its adhesion/biofilm properties.

Neutrophil activation and subsequent production of ROS were also enhanced by CF-ALF induced modifications on the *P*. *aeruginosa* cell envelope, which may contribute to the pathology of CF by impairing ALF clearance, interfering with innate immune functions, and overall driving neutrophilic inflammation. Because we found that so many hydrolases are upregulated in CF-ALF, the excessive neutrophil oxidative response to *P*. *aeruginosa* exposed to CF-ALF could be due to several factors, including the observed modifications on Psl cell surface expression, *P*. *aeruginosa* cell surface opsonization by CF-ALF components, and exposure of other components of the *P*. *aeruginosa* cell wall due to the actions of CF-ALF hydrolases. In this study we focused on Psl, as this, together with Pel and LPS, are major cell wall components of *P*. *aeruginosa*, and Psl is critical for biofilm formation and pathogenesis. In this context, the link between CF-ALF affecting the overall surface exposure of Psl and the increased production of ROS observed is in accordance with our previous studies showing that the presence of Psl in non-mucoid *P*. *aeruginosa* reduces the neutrophil production of ROS^15^. The complete absence of Psl in *P*. *aeruginosa* enhances serum complement opsonization of bacteria, thus favoring *P*. *aeruginosa* killing^15^. But this is not what we observed after *P*. *aeruginosa* is exposed to CF-ALF, where partial reduction of Psl from the cell surface resulted in a decrease of C3 opsonization but still triggered neutrophil ROS production. Therefore, this scenario defines CF-ALF as acting to eliminate Psl from the *P*. *aeruginosa* cell surface driving the immune response against the bacterium, but at the same time, originating an increase in ROS production that at the end may be detrimental for both, *P*. *aeruginosa* and the host due to tissue damage.

In summary, this study demonstrates that using a colorimetric approach we can identify the basal activity of several hydrolases in human CF-ALF, where we identified higher activity levels of several ALF hydrolases in CF patients compared to healthy individuals. Hydrolases with the most abundant catalytic activity in these samples were acid phosphatase, α-mannosidase, and some glycosidases, phospholipase C, and fatty acid esterases. These will be the focus of future studies to determine how these hydrolases may impact the outcome of microbial infections observed in CF patients. In the case of *P*. *aeruginosa*, these CF-ALF hydrolases reduce the cell surface exposure of Psl, which results in increase in ROS production by neutrophils upon contact with CF-ALF exposed *P*. *aeruginosa*. The dynamics of the lung environment in chronic diseases may dictate the establishment of a given infection in a determined time, where the *P*. *aeruginosa* encounter with ALF and the subsequent biological consequences in the establishment of the infections may provide answers to targeting specific ALF hydrolases to improve the quality of life for CF patients colonized with *P*. *aeruginosa*.

## Methods

### Patient Population

Bronchoalveolar lavage fluid (BALF) from CF individuals (n = 10) was obtained following the approved Nationwide Children’s Hospital (NCH) IRB for human subjects. There were 50:50 male/female with mean age of 17 years (range 7–30). The BALF was neutrophilic in nature with mean cell differential of 77% neutrophils, 18% macrophages, 3% lymphocytes, and 2% eosinophils. Control BALF from healthy human donors (n = 14, 50:50 male/female with mean age of 23, range 18–30 years of age) was performed as we previously described under a The Ohio State University (OSU) approved protocol for human subjects. All experiments were performed in accordance with the relevant guidelines and regulations. All BALF donors agreed to participate in this study and publication of this study by informed written consent.

### *P. aeruginosa* strains and growth conditions

*P*. *aeruginosa* strains were grown on Luria-Bertani (LBNS; tryptone 10 g per liter and yeast extract 5 g per liter without NaCl) at 37 °C for broth cultures. Solid medium (LANS) was prepared with the addition of 1.5% agar and also grown at 37 °C. The strains used in this study were *P*. *aeruginosa* PAO1, WFPA800 and PDO300^52–54^.

### Hydrolase activity determination in alveolar lining fluid (ALF)

ALF was obtained as we previously described^26^. Briefly, BALF was obtained by bronchoalveolar lavage from healthy and CF donors in 80 ml sterile 0.9% NaCl, 0.22-µM filtered to remove cells, depleted of lipids, concentrated 20-fold to achieve the ALF volume present within the human lungs^23^, and frozen at −80 °C until use for subsequent studies. A total of 14 different healthy ALF donors and 10 different CF ALF donors were used in these studies. A colorimetric method based on the cleavage of *p*-nitrophenol from a specific substrate upon enzymatic activity was used to measure the hydrolase activities present in the samples as we previously described^26^.

### Measuring growth rates with Biolog™

Biolog growth medium was prepared using LBNS and 1.2% of Biolog Dye A (Biolog, Hayward, CA). Fresh cultures of *P*. *aeruginosa* were grown to an O.D. A_600_ of 0.3. Up to 150 μl of this culture was combined with 750 μl of Biolog growth medium per sample and 100 μl, in triplicate, was placed into the Biolog plate wells (catalog #30311). The plate was incubated in the OmniLog® PM System (S/N #93171) at 37 °C for 24 hours using OmniLog® software analysis (*vr*. 1.2). The OmniLog® PM System generates a kinetic response curve for the growth or inhibition assays by monitoring and detecting changes in cellular respiration through measurement of spectroscopic change resulting from the reduction of the Biolog indicator dye. The changes in respiration reflected in “Biolog Units” correlate to growth of the particular organism. After 24 hours, data were compiled and exported from the OmniLog software using Microsoft Excel and graphically displayed using GraphPad Prism (*vr*. 5.03). In all cases, the mean signal values are presented.

### Polysaccharide immunoblotting and ELISA

Crude polysaccharide extracts were obtained by suspending either saline-, hydrolase- or CF-ALF-exposed bacteria in 250 μl of 500 mM EDTA and boiling for 5 min at 100 °C^55^. The suspension was then centrifuged at 20,800- × *g* for 3 min. Supernatants were then transferred to new tubes and treated with proteinase K stock solution (Qiagen, Valencia, CA) at a final concentration of 0.5 mg/ml for 60 min at 60 °C. The proteinase K was inactivated by heating at 80 °C for 30 min. Samples were stored at 4 °C prior to immunoblotting and ELISA.

For immunoblotting, 5 μl of crude polysaccharide extract was spotted onto Amersham Hybond-ECL nitrocellulose membrane (GE Healthcare Bio-Sciences, Pittsburgh, PA) and allowed to air dry. The membrane was blocked with 5% non-fat dry milk in TBST (20 mM Tris, 137 mM NaCl, 0.1% Tween 20, pH 7.6) for one hour at room temperature with agitation followed by probing with α-Psl polyclonal antibody (diluted 1:25,000 in TBST) as previously describe^34^. For quantification of the Psl in the polysaccharide extracts, ELISA was performed as previously described^34^ using a α-Psl polyclonal antibody (diluted 1:25,000 in PBS).

### Monosaccharide Analysis

Samples were hydrolyzed with 2 M trifluoroacetic acid and converted to alditol acetates by reducing with NaBD_4_ in NH_4_OH followed by acetylation using acetic anhydride^56^. Alditols were analyzed on a Thermo Scientific gas chromatography-mass spectrometer model DSQ-II (Bruker-Daltonics, Billerica, MA), using *scyllo*-inositol as an internal standard, as previously described^26^.

### Microtiter dish attachment assay

Attachment of *P*. *aeruginosa* to an abiotic surface was performed as previously described (39). Briefly, bacteria were grown to mid-log (O.D. A_600_ of 0.5) and treated with CF ALF for one hour at 37 °C. After treatment, the bacteria were pelleted by centrifugation and washed three times in 0.9% NaCl. After the final wash the bacteria were suspend in LBNS and 100 μL of the pellet was added to wells of a 96-well PVC microtiter plate (Costar Inc., Corning, NY) for 30 minutes at room temperature. Non-adherent bacteria were removed by washing thoroughly in water. Adherent bacteria were stained with 125 μL of 0.1% crystal violet per well and incubated at room temperature for fifteen minutes. The plate was washed in water and the bound crystal violet was solubilized in 150 μL of 95% ethanol for fifteen minutes at room temperature. The solubilized stain was transferred to a 96-well microtiter plate and the absorbance measured at O.D. A_540_.

### *P. aeruginosa* C3 opsonization

C3 deposition in *P*. *aeruginosa* was determined as previously described^15^. Briefly, *P*. *aeruginosa* (1.7 × 10^8^ bacteria per mL) was opsonized with 20% pooled human serum at 37 °C for 15 minutes with gentle rocking. Bacteria were centrifuged at 21,000- × *g* for 5 minutes at 4 °C, washed twice with cold PBS on ice, and fixed with cold 2% paraformaldehyde for 10 minutes on ice. Bacteria were then stained with a mouse anti-human activated C3 antibody (Santa Cruz Biotechnology Inc., #sc47687, Dallas, Texas) or a PE-conjugated mouse IgG1 k isotype control isotype (BD, #555749, San Jose, CA) on ice in the dark for 20 minutes. C3-stained cells were subsequently stained with a PE-conjugated goat anti-mouse antibody (Abcam, #ab7002, Cambridge, MA) on ice in the dark for 20 minutes. Samples were analyzed using BD FACS CANTO II flow cytometer (BD Biosciences, San Jose, CA) by reading >10,000 events per sample, and data analyzed using FlowJo *Vr*. 9.7.6 Software.

### Neutrophil isolation, activation status and oxidative burst studies

Human neutrophils were obtained from healthy adult donors as we previously described^27^ using an approved IRB protocol (2009H0314) at The Ohio State University. Surface expression of activation and degranulation markers on human neutrophils was assessed by flow cytometry as we previously described^27^. Briefly, neutrophils were infected with healthy ALF-exposed *P*. *aeruginosa* or CF-ALF-exposed *P*. *aeruginosa* at 37 °C, 5% CO_2_ for 30 min. After infection, neutrophils were stained with antibodies specific for cell-surface markers (BD PE-mouse anti-human CD63-Ab, BD PerCP/Cy5.5-mouse anti-human CD66b-Ab, and BD FITC-mouse anti-human CD11b-Ab; BD Biosciences, Franklin Lakes, NJ) for 30 min on ice in dark conditions. Neutrophil surface expression of CD63 and CD66b, markers of primary and secondary granules, respectively, and CD11b expression were assessed by flow cytometry (≥10,000 events). All appropriate isotype controls were included (BD PE-mouse IgG1 ĸ isotype control-Ab, BD PerCP/Cy5.5-mouse IgM ĸ isotype control-Ab and BD FITC-mouse IgG1 ĸ isotype control-Ab). Samples were read on a BD FACS CANTO II flow cytometer (BD Biosciences, San Jose, CA) and data were analyzed using BD FACS Diva and FlowJo *vr*. 9.7.6 Software.

The luminol chemiluminescence assay was used to detect intracellular and extracellular reactive oxygen species (total ROS) generated by neutrophils upon interaction with *P*. *aeruginosa* ^57^. Actively growing *P*. *aeruginosa* (non-mucoid *P*. *aeruginosa* PAO1 and mucoid *P*. *aeruginosa* PDO300) were diluted to a final concentration of 3 × 10^7^/ml, washed three times with sterile HBSS and exposed with either sterile 0.9% NaCl, CF-ALF or H-ALF for 1 h at 37 °C, 5% CO_2_. Bacteria were then washed three times and further opsonized with 20% pooled fresh normal human serum for 30 min at 37 °C. Following opsonization, bacteria were washed and used for infections. ROS production was determined via a luminescent assay. Phorphol 12-myristate 13-acetate (PMA) was used at 10 ng/ml as a positive control for the generation of ROS, while non-opsonized *P*. *aeruginosa* was used as a negative control. Neutrophils were diluted to a final concentration of 4 × 10^6^/ml in HBSS containing 50 mM luminol and seeded onto a 96-well microtiter plate. The prepared bacteria were added to the neutrophils at an MOI of 50:1. ROS generation was measured at regular intervals over one hour by luminescence using a SpectraMax M5 plate reader (Molecular Devices LLC, Sunnyvale, CA) Relative light units (RLU) were plotted as a function of time to evaluate chemiluminescence (CL) rate^58^.

### Statistics

Statistical analyses were performed using GraphPad 4.0/5.0 Prism software to determine the statistical significance between the means for 2 experimental groups using an unpaired, 2-tailed Student’s t test. Differences were considered statistically significant at a *^,§^ *p* < 0.05; **^,§§^ *p* < 0.005; ***^,§§§^ *p* < 0.0005.

## References

[1] Morrison AJ, Wenzel RP (1984). Epidemiology of infections due to *Pseudomonas aeruginosa*. Rev. Infect. Dis..

[2] Rutala WA, Weber DJ (1997). Water as a reservoir of nosocomial pathogens. Infect. Control Hosp. Epidemiol..

[3] Rowe SM, Miller S, Sorscher EJ (2005). Cystic fibrosis. N. Engl. J. Med..

[4] Folkesson A (2012). Adaptation of *Pseudomonas aeruginosa* to the cystic fibrosis airway: an evolutionary perspective. Nat. Rev. Microbiol..

[5] Hoiby N (2011). The clinical impact of bacterial biofilms. Int. J. Oral Sci..

[6] Singh PK (2000). Quorum-sensing signals indicate that cystic fibrosis lungs are infected with bacterial biofilms. Nature..

[7] Lam J, Chan R, Lam K, Costerton JW (1980). Production of mucoid microcolonies by *Pseudomonas aeruginosa* within infected lungs in cystic fibrosis. Infect. Immun..

[8] O’toole GA (2003). To build a biofilm. J. Bacteriol..

[9] Parsek MR, Singh PK (2003). Bacterial biofilms: An emerging link to disease pathogenesis. Annu. Rev. Microbiol..

[10] Stoodley P, Sauer K, Davies DG, Costerton JW (2002). Biofilms as complex differentiated communities. Annu. Rev. Microbiol..

[11] Ryder C, Byrd M, Wozniak DJ (2007). Role of polysaccharides in *Pseudomonas aeruginosa* biofilm development. Curr. Opin. Microbiol..

[12] May TB (1991). Alginate synthesis by *Pseudomonas aeruginosa*: A key pathogenic factor in chronic pulmonary infections of cystic fibrosis patients. Clin. Microbiol. Rev..

[13] Wozniak DJ (2003). Alginate is not a significant component of the extracellular polysaccharide matrix of PA14 and PAO1 *Pseudomonas aeruginosa* biofilms. Proc. Natl. Acad. Sci. USA.

[14] Colvin KM (2011). The pel polysaccharide can serve a structural and protective role in the biofilm matrix of *Pseudomonas aeruginosa*. PLoS. Pathog..

[15] Mishra M (2012). *Pseudomonas aeruginosa* Psl polysaccharide reduces neutrophil phagocytosis and the oxidative response by limiting complement-mediated opsonization. Cell Microbiol.

[16] Colvin KM (2012). The Pel and Psl polysaccharides provide *Pseudomonas aeruginosa* structural redundancy within the biofilm matrix. Environ. Microbiol..

[17] Ghafoor A, Hay ID, Rehm BH (2011). Role of exopolysaccharides in *Pseudomonas aeruginosa* biofilm formation and architecture. Appl. Environ. Microbiol..

[18] Yang L (2011). Distinct roles of extracellular polymeric substances in *Pseudomonas aeruginosa* biofilm development. Environ. Microbiol.

[19] Jorth P (2015). Regional isolation drives bacterial diversification within cystic fibrosis lungs. Cell Host Microbe..

[20] Ulrich M (2010). Alveolar inflammation in cystic fibrosis. J. Cyst. Fibros..

[21] Ochs M (2004). The number of alveoli in the human lung. Am. J. Respir. Crit Care Med..

[22] Torrelles, J. B. & Schlesinger, L. S. Integrating lung physiology, immunology, and tuberculosis. *Trends Microbiol*. pii: S0966-842X(17)30070-7, doi:10.1016/j.tim.2017.03.007 [Epub ahead of print] (2017). 10.1016/j.tim.2017.03.007 PMC5522344 28366292

[23] Notter, R. H. Lung surfactants: Basic science and clinical applications (ed. Dekker, M.) 1–444 (New York, NY, 2000).

[24] Nicod LP (2005). Lung defenses: An overview. Eur. Resp. Rev..

[25] Sasindran J, Torrelles JB (2011). *Mycobacterium tuberculosis* infection and inflammation: What is beneficial for the host and for the bacterium?. Front. Microbiol..

[26] Arcos J (2011). Human lung hydrolases delineate *Mycobacterium tuberculosis*-macrophage interactions and the capacity to control infection. J. Immunol..

[27] Arcos J (2015). Lung mucosa lining fluid modifies *Mycobacterium tuberculosis* to reprogram human neutrophil killing mechanisms. J. Infect. Dis..

[28] Arcos, J. *et al*. *Mycobacterium tuberculosis* cell wall released fragments by the action of the human lung mucosa modulate macrophages to control infection in an IL-10-dependent manner. *Mucosal Immunol*., doi:10.1038/mi.2016.115 [Epub ahead of print] (2017). 10.1038/mi.2016.115 PMC5479761 28000679

[29] Scordo J (2017). *Mycobacterium tuberculosis* cell wall fragments released upon bacterial contact with the human lung mucosa alter the neutrophil response to infection. Front Immunol..

[30] Mann EE, Wozniak DJ (2012). Pseudomonas biofilm matrix composition and niche biology. FEMS Microbiol. Rev..

[31] Ma L, Lu H, Sprinkle A, Parsek MR, Wozniak DJ (2007). *Pseudomonas aeruginosa* Psl is a galactose- and mannose-rich exopolysaccharide. J. Bacteriol..

[32] Zhao K (2013). Psl trails guide exploration and microcolony formation in *Pseudomonas aeruginosa* biofilms. Nature..

[33] Ames BN (1966). Assay of inorganic phosphate, total phosphate and phosphatases. Methods Enzymol.

[34] Byrd MS (2009). Genetic and biochemical analyses of the *Pseudomonas aeruginosa* Psl exopolysaccharide reveal overlapping roles for polysaccharide synthesis enzymes in Psl and LPS production. Mol. Microbiol..

[35] Griese M (1999). Pulmonary surfactant in health and human lung diseases: State of the art. Eur. Respir. J..

[36] Griese M, Birrer P, Demirsoy A (1997). Pulmonary surfactant in cystic fibrosis. Eur. Respir. J.

[37] Gifford AM, Chalmers JD (2014). The role of neutrophils in cystic fibrosis. Curr. Opin. Hematol..

[38] Kehely A, Moss DW (1992). Circulating levels of tartrate-resistant acid phosphatase in macrophage-activated lung disease. Ann. Clin. Biochem..

[39] Nanjundan M, Possmayer F (2003). Pulmonary phosphatidic acid phosphatase and lipid phosphate phosphohydrolase. Am. J Physiol Lung Cell Mol. Physiol..

[40] Benson BJ (1980). Properties of an acid phosphatase in pulmonary surfactant. Proc. Natl. Acad. Sci. USA.

[41] Hite RD (2005). Lysophospholipid generation and phosphatidylglycerol depletion in phospholipase A(2)-mediated surfactant dysfunction. Am. J. Physiol Lung Cell Mol. Physiol..

[42] de Vries AC, Schram AW, Tager JM, Batenburg JJ, van Golde LM (1985). A specific acid alpha-glucosidase in lamellar bodies of the human lung. Biochim. Biophys. Acta..

[43] Pezzulo AA (2012). Reduced airway surface pH impairs bacterial killing in the porcine cystic fibrosis lung. Nature..

[44] Terry JM, Pina SE, Mattingly SJ (1992). Role of energy metabolism in conversion of nonmucoid *Pseudomonas aeruginosa* to the mucoid phenotype. Infect. Immun..

[45] Terry JM, Pina SE, Mattingly SJ (1991). Environmental conditions which influence mucoid conversion *Pseudomonas aeruginosa* PAO1. Infect. Immun..

[46] Temple, L., Sage, A., Schweizer, H. & Phibbs, P. Carbohydrate metabolism in *Pseudomonas aeruginosa*. In *Pseudomonas* (ed. Montie, T. C.) 35–72 (Plenum Press, New York, NY, 1998).

[47] Rocchetta HL, Burrows LL, Pacan JC, Lam JS (1998). Three rhamnosyltransferases responsible for assembly of the A-band D-rhamnan polysaccharide in *Pseudomonas aeruginosa*: A fourth transferase, WbpL, is required for the initiation of both A-band and B-band lipopolysaccharide synthesis. Mol. Microbiol..

[48] Soberon-Chavez G, Lepine F, Deziel E (2005). Production of rhamnolipids by *Pseudomonas aeruginosa*. Appl. Microbiol. Biotechnol..

[49] Almeida RA, Wannemuehler MJ, Rosenbusch RF (1992). Interaction of *Mycoplasma dispar* with bovine alveolar macrophages. Infect. Immun..

[50] Friedman L, Kolter R (2004). Two genetic loci produce distinct carbohydrate-rich structural components of the *Pseudomonas aeruginosa* biofilm matrix. J. Bacteriol..

[51] Matsukawa M, Greenberg EP (2004). Putative exopolysaccharide synthesis genes influence Pseudomonas aeruginosa biofilm development. J. Bacteriol..

[52] Holloway BW (1955). Genetic recombination in *Pseudomonas aeruginosa*. J. Gen. Microbiol.

[53] Mathee K, McPherson CJ, Ohman DE (1997). Posttranslational control of the algT (algU)-encoded sigma22 for expression of the alginate regulon in *Pseudomonas aeruginosa* and localization of its antagonist proteins MucA and MucB (AlgN). J. Bacteriol..

[54] Ma L, Jackson KD, Landry RM, Parsek MR, Wozniak DJ (2006). Analysis of *Pseudomonas aeruginosa* conditional psl variants reveals roles for the psl polysaccharide in adhesion and maintaining biofilm structure postattachment. J. Bacteriol..

[55] Parise G, Mishra M, Itoh Y, Romeo T, Deora R (2007). Role of a putative polysaccharide locus in Bordetella biofilm development. J. Bacteriol..

[56] McNeil M, Chatterjee D, Hunter SW, Brennan PJ (1989). Mycobacterial glycolipids: Isolation, structures, antigenicity, and synthesis of neoantigens. Methods Enzymol..

[57] Engels W, Endert J, Kamps MA, van Boven CP (1985). Role of lipopolysaccharide in opsonization and phagocytosis of *Pseudomonas aeruginosa*. Infect. Immun..

[58] Mohapatra NP (2010). Francisella acid phosphatases inactivate the NADPH oxidase in human phagocytes. J. Immunol..

